# In Vitro Investigation of Thiol-Functionalized Cellulose Nanofibrils as a Chronic Wound Environment Modulator

**DOI:** 10.3390/polym13020249

**Published:** 2021-01-13

**Authors:** Anna Blasi-Romero, Carlos Palo-Nieto, Corine Sandström, Jonas Lindh, Maria Strømme, Natalia Ferraz

**Affiliations:** 1 Nanotechnology and Functional Materials, Department of Material Science and Engineering, Uppsala University, Box 35, 75103 Uppsala, Sweden; anna.blasi@angstrom.uu.se (A.B.-R.); carlos.nieto@angstrom.uu.se (C.P.-N.); jonas.lindh@angstrom.uu.se (J.L.); maria.stromme@angstrom.uu.se (M.S.); 2 Department of Molecular Sciences, Swedish University of Agricultural Sciences, Box 7015, 75007 Uppsala, Sweden; corine.sandstrom@slu.se

**Keywords:** nanocellulose, wound healing, proteases, reactive oxygen species, cysteine, antioxidant properties

## Abstract

There is currently a huge need for new, improved therapeutic approaches for the treatment of chronic wounds. One promising strategy is to develop wound dressings capable of modulating the chronic wound environment (e.g., by controlling the high levels of reactive oxygen species (ROS) and proteases). Here, we selected the thiol-containing amino acid cysteine to endow wood-derived cellulose nanofibrils (CNF) with bioactivity toward the modulation of ROS levels and protease activity. Cysteine was covalently incorporated into CNF and the functionalized material, herein referred as cys-CNF, was characterized in terms of chemical structure, degree of substitution, radical scavenging capacity, and inhibition of protease activity. The stability of the thiol groups was evaluated over time, and an in vitro cytotoxicity study with human dermal fibroblasts was performed to evaluate the safety profile of cys-CNF. Results showed that cys-CNF was able to efficiently control the activity of the metalloprotease collagenase and to inhibit the free radical DPPH (1,1-Diphenyl-2-picrylhydrazyl radical), activities that were correlated with the presence of free thiol groups on the nanofibers. The stability study showed that the reactivity of the thiol groups challenged the bioactivity over time. Nevertheless, preparing the material as an aerogel and storing it in an inert atmosphere were shown to be valid approaches to increase the stability of the thiol groups in cys-CNF. No signs of toxicity were observed on the dermal fibroblasts when exposed to cys-CNF (concentration range 0.1–0.5 mg/mL). The present work highlights cys-CNF as a promising novel material for the development of bioactive wound dressings for the treatment of chronic wounds.

## 1. Introduction

Cutaneous wound healing is a well-orchestrated process consisting of four overlapping phases: hemostasis, inflammation, proliferation, and remodeling. Most skin wounds heal within 2–3 weeks, however, imbalances in the wound healing phases can lead to non-healing chronic wounds [1]. Chronic wounds remain in the inflammatory phase, where elevated levels of reactive oxygen species (ROS), pro-inflammatory cytokines, and degradative proteases result in reduced concentrations of growth factors and proteinase inhibitors, and an imbalance in the wound equilibrium [2]. Diabetes and vascular insufficiency are the main underlying causes of non-healing wounds, with systemic factors such as advanced age or a compromised immune system also contributing to poor wound-healing. The incidence of chronic wounds has dramatically increased in recent years, now reaching epidemic proportions [3]. Presently, most of the widely used wound dressings only provide an optimal local healing milieu (i.e., they protect the wound from further trauma, provide moisture, and adsorb excess exudate) [4]. To improve the treatment of chronic wounds, it has been proposed that new types of wound-healing therapies will be required, with the aim of obtaining bioactive dressings that stimulate local cells to migrate and proliferate, stimulate, and guide extracellular matrix deposition, modulate protease activity, and/or neutralize free radicals [4].

Nanocellulose is emerging as an interesting material for biomedical applications such as tissue engineering, drug delivery, and wound care [5,6]. Nanocellulose consists of cellulose fibrils or crystallites with dimensions in the nanoscale and is obtained from a diversity of sources such as wood, algae, bacteria, and tunicates [7]. Wood-derived cellulose nanofibrils (CNF) comprise individual fibrils that are 2–10 nm in diameter and several micrometers in length, typically forming 20–60 nm thick aggregates [8]. Characteristics such as being a renewable material, not being of animal origin, having tuneable properties in terms of surface chemistry, aspect ratio and form, and being produced in a process that can easily be scaled-up industrially have contributed to the increased interest in using CNF for biomedical applications [5,9]. CNF have been described as a promising material for the treatment of wounds [5,10,11]. Authors have highlighted the physicochemical properties of the material and the absence of cytotoxicity as beneficial characteristics for employing CNF films, aerogels, and gel suspensions as wound dressings [12,13,14]. Moreover, clinical studies have shown encouraging results when using CNF films on skin graft donor sites [15,16]. Our group has demonstrated the in vitro and in vivo wound healing properties of an ion-crosslinked CNF hydrogel [17,18,19] together with the evaluation of its antibacterial properties [20] and the possibility of using the hydrogel as a drug delivery dressing [21].

In the present work, we selected the amino acid cysteine as an active molecule to endow CNF with bioactivity and for the first time investigate the potential of the functionalized CNF material to modulate the chronic wound environment in vitro. It is believed that addressing the biochemical imbalances typically found in chronic wounds will aid in the resolution of these hard-to-heal wounds. Thus, by targeting the excess of ROS and the high level of metalloprotease activity, it may be possible to restore basal levels of growth factors, promote the restitution of the proper balance between degradation and formation of new tissue, and help overcome the prolonged inflammatory phase observed in chronic wounds [22,23]. The radical scavenging properties of cysteine and its metal chelating ability [24,25], make this amino acid an excellent candidate molecule to be used in the development of wound dressings capable of interacting with the biochemical environment of chronic wounds.

In this study, cysteine was covalently incorporated into CNF and the functionalized material was characterized in terms of chemical structure and degree of substitution. The radical scavenging properties and the capacity of the material to inhibit metalloprotease activity were investigated. Furthermore, the stability of the thiol groups in cys-CNF and its bioactivity were evaluated over time. Finally, the safety profile of the thiol-functionalized CNF was investigated in an in vitro cytotoxicity study with human dermal fibroblasts.

## 2. Materials and Methods

### 2.1. Chemicals and Reagents

Carboxylated-CNF, provided by RISE Bioeconomy (Stockholm, Sweden), was produced from commercial never-dried bleached sulfite softwood dissolving pulp (lignin content < 1.5%, xylose < 1.7%, mannose < 1.8%, Domsjö Fabriker AB, Örnsköldsvik, Sweden) by 2,2,6,6-tetramethylpiperidine-1-oxyl (TEMPO)-mediated oxidation. L-cysteine methyl ester hydrochloride, *N*-(3-dimethyolaminopropyl)-*N*′-ethylcarbodiimide hydrochloride (EDC), *N*-hydroxysuccinimide (NHS), collagenase from *Clostridium histolyticum,* and calcein-AM were obtained from Sigma Aldrich (St. Louis, MO, USA). Fetal bovine serum (FBS), penicillinin, streptomycin, 5,5-dithio-bis-(2-nitrobenzoic acid) (DTNB), EnzChek™ Gelatinase/Collagenase Assay Kit, Pierce ^TM^ Bradford Protein Assay Kit, DMEM:F12 medium, fetal bovine serum, and presto blue cell viability reagent were obtained from ThermoFisher Scientific (Waltham, MA, USA). 1,1-Diphenyl-2-picrylhydrazyl radical (DPPH) was purchased from Santa Cruz Biotechnology (Dallas, TX, USA).

### 2.2. Covalent Incorporation of Methyl Cysteine to Cellulose Nanofibrils

**Figure 1:**
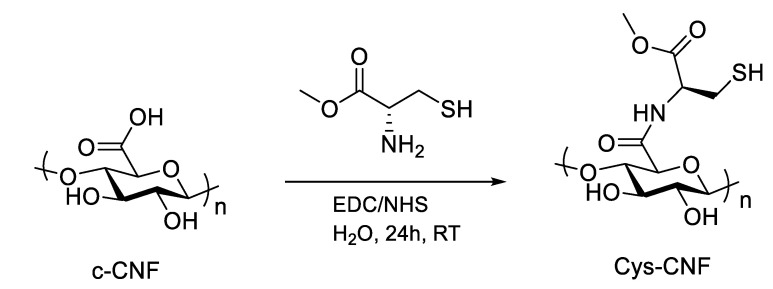
Reaction scheme of the chemical modification of carboxylated cellulose nanofibrils (c- CNF) with L-cysteine methyl ester to obtain cysteine-functionalized CNF (cys-CNF).

L-cysteine methyl ester was covalently incorporated to carboxylated CNF (c-CNF, carboxyl group content 1300 μmol/g dry CNF) via EDC/NHS coupling (Figure 1). Briefly, 100 g of c-CNF suspension corresponding to 1 g of dried cellulose was dispersed in 50 mL of deionized water and 5 mL of aqueous solution containing 180 mg of NHS and 290 mg of EDC (corresponding to 1.2 mmol of NHS/EDC per mol of carboxyl group in CNF) were added under stirring, followed by the addition of 10 mL of L-cysteine methyl ester aqueous solution. Two different molar ratios cysteine:COOH were used in the synthesis, corresponding to 6 and 20 equivalents of cysteine with respect to the carboxyl groups in c-CNF. The reaction mixture was stirred for 24 h at pH 5 and room temperature. For purification, three cycles of centrifugation (30 min at 3380× *g*) were carried out, followed by dialysis against deionized water until the conductivity in water was < 0.005 mS/cm^2^.

### 2.3. Material Characterization

#### 2.3.1. Characterization of the Molecular Structure

The chemical structure of cys-CNF was characterized by solid-state nuclear magnetic resonance (NMR). The NMR spectra were obtained on a Bruker Avance III 600 MHz spectrometer (Bruker, Billerica, MA, USA) using a double-resonance 4 mm (^1^H&^19^F)/(^15^N-^31^P) CP-MAS probe and 4 mm ZrO_2_ rotors. The ^13^C cross-polarization (CP) magic angle spinning (MAS) NMR spectra were recorded at a spinning frequency of 12 KHz, a contact time of 1–2 ms and a repetition delay of 3–5 s. The experiments were performed at 298 K.

#### 2.3.2. Degree of Substitution

The cysteine content of cys-CNF was determined by elemental analysis of total sulfur content. For this, cys-CNF freeze-dried samples were submitted to MEDAC Ltd., analytical and chemical consultancy services (Cobham, UK), where a Thermo FlashEA^R^ 1112 instrument was used for sulfur quantification. The weight percentage of sulfur in cys-CNF was converted to mmol cysteine/g CNF [26] and the degree of substitution (DS) was calculated as the cysteine content (mmol/g CNF) per number of carboxyl groups in the starting material c-CNF (1.3 mmol/g CNF).

#### 2.3.3. Thiol Group Content

The thiol group content was quantified by the Ellman assay. Briefly, 2.5 mL of reaction buffer (0.1 M sodium phosphate, pH 8.0, containing 1 mM EDTA) was mixed with 250 µL of cys-CNF suspension (0.05 wt%) and 50 µL of DTNB solution (4 mg/mL in reaction buffer). After 15 min, the absorbance at 412 nm was measured using a spectrophotometer (UV-1800, Shimadzu, Kyoto, Japan). The number of thiol groups in the CNF samples was calculated using a L-cysteine methyl ester standard curve and expressed as mmol SH/g CNF.

### 2.4. Radical Scavenging Capacity

Free radical scavenging capacity was evaluated with DPPH by measuring the decrease in absorbance of the radical at 517 nm after incubation with the CNF materials. In brief, 1.2 mL of CNF suspension in water (containing 0.5 mg, 2 mg, and 5 mg of dry CNF materials) was mixed with 10.8 mL of DPPH (0.2 mM in methanol). After 90 min incubation time protected from direct light, reaction tubes were centrifuged to spin down the CNF materials and the absorbance of the supernatant was measured at 517 nm with a plate reader (TECAN infinite M200, Männedorf, Switzerland). Radical scavenging capacity was expressed as percentage of DPPH inhibition according to Equation (1):

(1) $$\mathrm{Radical}\;\mathrm{scavenging}\;\mathrm{capacity}\;\left(\;\%\;\mathrm{DPPH}\;\mathrm{inhibition}\right)=\left(1\;-\;\frac{A_{S}}{A_{R}}\right)*\;100,$$

where A_S_ is the absorbance at 517 nm of the DPPH solution after incubation with the CNF samples and A_R_ is the absorbance at 517 nm of the DPPH reference solution (i.e., incubated alone).

### 2.5. Evaluation of the Material Interactions with the Metalloprotease Collagenase

Collagenase from *Clostridium histolyticum* was selected as a model protein to investigate the interaction of cys-CNF with metalloproteases. Aqueous suspensions (0.7 mL) of the CNF materials containing 0.1 mg, 0.2 mg, and 3.5 mg of dry cellulose were mixed with collagenase in reaction buffer (0.5 M Tris-HCl, 1.5 M NaCl, 50 mM CaCl_2_, and 2 mM sodium azide, pH 7.6) at a final protein concentration of 10 μg/mL in a final volume of 1.5 mL. The mixture was incubated for 24 h at 37 °C under agitation (400 rpm). Afterward, reaction tubes were centrifuged to spin down the fibers and the supernatant was collected to determine the collagenase activity and concentration.

#### 2.5.1. Evaluation of Protease Inhibition

The EnzChek^TM^ Collagenase Assay Kit was used to quantify the activity of collagenase after incubation with the materials. The assay was performed as indicated by the manufacturer. Briefly, 20 µL of fluorescein conjugate-gelatin (DQ^TM^ Gelatin, 0.5 mg/mL) was added to 100 µL of reaction buffer (0.5 M Tris-HCl, 1.5 M NaCl, 50 mM CaCl_2_, and 2 mM sodium azide, pH 7.6) mixed with 80 µL of CNF-incubated collagenase solution. The increase in fluorescence signal (excitation/emission at 485/530 nm), as a result of substrate cleavage by the collagenase, was measured from minute 0 to 120 with a plate reader (TECAN infinite M200, Männedorf, Switzerland). To calculate the inhibition of collagenase activity, the 30 min time point was selected and the fluorescence intensity of the CNF-incubated collagenase samples was compared to the fluorescence intensity of the collagenase control according to Equation (2):

(2) $$\mathrm{Inhibition}\;\mathrm{collagenase}\;\mathrm{activity}\;\left(\%\right)=\;\left(1\;-\;\frac{F_{\mathrm{sample}}}{F_{\mathrm{control}}}\right)*\;100,$$

where F is fluorescence intensity (a.u.) at the 30 min time point.

#### 2.5.2. Evaluation of Protease Entrapment

The Bradford assay was used for protein quantification. Simply, 100 µL of CNF-incubated collagenase was mixed with 100 µL of Bradford reagent in a transparent 96 well plate. Following the protocol from the manufacturer, the plate was shacked for 30 s and, after 10 min incubation at room temperature, absorbance was measured at 595 nm with a plate reader (TECAN infinite M200, Männedorf, Switzerland). Protein entrapment was expressed as percentage of collagenase in solution after incubation with CNF over the content of the protein in the control solution, as expressed in Equation (3):

(3) $$\mathrm{Protein}\;\mathrm{Entrapment}\;\left(\%\right)=\;\left(1\;-\;\frac{A_{S}}{A_{R}}\right)*100$$

where A_S_ and A_R_ are the absorbance values at 595 nm of the sample and of the control solution, respectively.

#### 2.5.3. Zn^2+^ Binding Assay

With similar conditions as in the collagenase inhibition experiment, water suspension of the CNF materials (5 mg dry content) were mixed and incubated with 0.1 mM zinc acetate in a final volume of 3 mL. The mixture was left in a shaking plate at 37 °C for 24 h. Then, fibers were spun down and the zincon assay was performed to quantify the remaining zinc in the supernatant. Briefly, 25 µL of sample (supernatant) was mixed with 950 µL of 53 mM borate buffer, pH 9; and 25 µL of 1.6 mM zincon (2-carboxy-2′-hydroxy-5′-sulfoformazyl-benzene) solution were added to the mixture. The absorbance at 620 nm was measured 5 min later. Zinc content was expressed as a percentage of the reference solution (i.e., where the CNF suspension was replaced by water).

### 2.6. Material Stability over Time

The thiol content and the radical scavenging properties of the cys-CNF suspension were measured by the Ellman assay and the DPPH assay, respectively, over a time period of 30 days to evaluate the stability of the thiol group in the cys-CNF suspension and the correlation to its radical scavenging capacity. The material was kept at 4 °C in three different storage conditions: suspensions in a closed container, aerogels prepared by freeze-drying, and suspensions stored under an inert atmosphere. The Ellman assay and the DPPH assay were conducted following the protocols described in Section 2.3 and Section 2.4, respectively.

### 2.7. Cell Studies

#### 2.7.1. Cell Culture

Adult human dermal fibroblasts (hDF) (primary cells from the European Collection of Authenticated Cell Cultures (ECACC, Salisbury, England) were cultured in DMEM-F12 medium supplemented with 10% *v/v* fetal bovine serum, 100 U/mL penicillin, and 100 μg/mL streptomycin. Cells were cultured at 37 °C and 5% CO_2_ in a humidified atmosphere and passaged at 80% confluency.

#### 2.7.2. Cytotoxicity Assessment

The cytotoxicity of cys-CNF was assessed by exposing hDF monolayers to suspensions of cys-CNF (1:20). Concentrations from 0.1 to 1 mg/mL in cell culture medium were prepared from 10 mg/mL stock suspension in water the day of the experiment. A control experiment showed that the dilution of cell culture medium due to the water content of the CNF stock suspension did not significantly affect the cell viability.

hDF cells (passage numbers 10–16) were seeded in a 96 well-plate at a density of 4.8 × 10^3^ cells/well and after 24 h of culture, they were exposed to the cys-CNF suspensions (200 µL/well) and cultured for another 24 h. Non-treated cells were used as the negative control and cells exposed to 5% DMSO in cell culture medium were the positive control. c-CNF was also included in the toxicity assessment in the same concentration range as cys-CNF.

Cellular metabolic activity was measured with the presto blue (PB) assay after 24 h of exposure to the CNF materials. Cell culture media were removed from the cell culture wells and cells were carefully washed with warm PBS. Thereafter, 200 µL of PB reagent diluted 1:10 in cell culture media were added to each well and incubated for 90 min at 37 °C, and 5% CO_2_ in a humidified atmosphere. Aliquots of 100 µL were transferred to a black 96-well plate and fluorescence was measured at 560 nm excitation and 590 nm emission wavelengths using a plate reader (Tecan infinite M200, Männedorf, Switzerland). Cell metabolic activity was used as an indicator of cell viability and the results were expressed as the percentage of the negative control. The interaction of the CNF samples with the PB reagent was evaluated by applying the same protocol as above, but in the absence of cells and the results showed no interference of the samples with the PB assay.

Cell viability and morphology were also assessed by microscopic observations of calcein-AM stained cells. Briefly, cell media were removed from the wells, and 100 µL of calcein-AM (0.1% *v/v* in PBS) were added to each well. After 15 min incubation time in the cell incubator, cells were imaged with a fluorescence microscope (Nikon Eclipse TE2000-U, Tokyo, Japan).

### 2.8. Statistical Analysis

All data are presented as mean ± standard error of the mean from at least three independent experiments, each with triplicate sampling. Statistical analyses were performed using GraphPad Prism software. Differences between groups were evaluated by one-way analysis of variance (ANOVA), followed by Dunnett’s test for pairwise comparisons. A *t*-test was used for paired comparison of the two treatment groups. The differences were considered statistically significant when the *p* value was < 0.05.

## 3. Results and Discussion

### 3.1. Material Characterization

The broad spectrum of possible chemical modifications of cellulose opens up the possibility of endowing the material with novel properties. Typically, surface chemical modifications of cellulose are employed during the production of CNF to introduce charged groups and facilitate the defibrillation process. Furthermore, post-production chemical modifications of CNF can take advantage of these introduced charged groups to explore new functionalization and obtain CNF materials with added value [27]. In the present work, we investigated the possibility of obtaining a bioactive CNF material by thiol-functionalization of the fibrils, with the ultimate aim of modulating the high levels of ROS and metalloproteinases found in hard-to-heal wounds. The carboxyl groups in the starting CNF, introduced by TEMPO-oxidation pretreatment of the wood pulp, were targeted to react with the amine groups of the thiol-amino acid cysteine via the EDC/NHS-mediated coupling.

**Figure 2:**
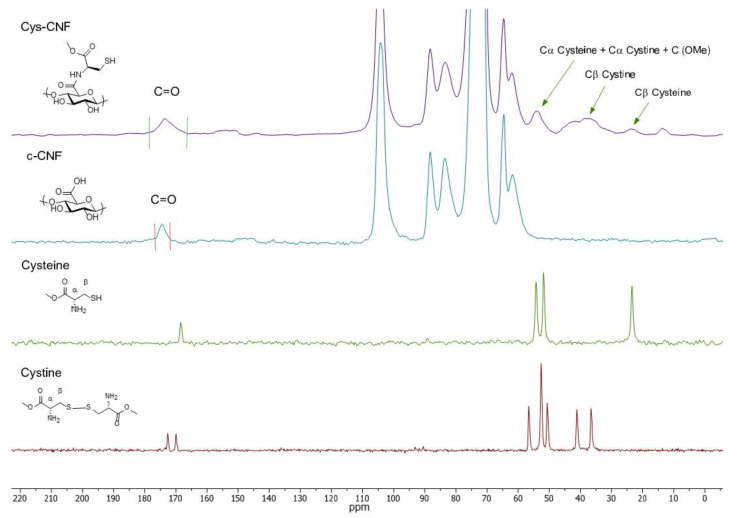
Solid-state nuclear magnetic resonance (NMR) spectra of cys-CNF and the references c-NFC, cysteine, and cystine.

The covalent incorporation of cysteine to CNF was investigated by CP/MAS ^13^C-NMR spectroscopy. The ^13^C-NMR spectrum of cys-CNF is displayed in Figure 2, together with the spectrum of c-CNF, cysteine, and cystine (cysteine oxidized dimer) as references. The signals at 24 ppm in the cys-CNF spectrum could be attributed to the Cβ in cysteine while the signals around 37 and 43 ppm were assigned to the methylene groups in cystine. The signals from the Cα and OMe carbons in cysteine and cystine were found to merge around 54 ppm. The broadening of the signals from cysteine and cystine in cys-CNF indicates that these two compounds interacted with c-CNF, reducing the motions of the carbons. Close inspection of the 170–180 ppm region showed a broad shoulder upfield to the signal of the carboxyl resonance of CNF. This was tentatively assigned to the carbonyl of the ester in cysteine and cystine and to the amide linkages formed between the amino acid and c-CNF. Similar effects have been shown in previous studies on cellulose-amino acid copolymers [28,29]. Altogether, the analysis of the solid-state NMR spectra indicated the presence of covalently bound cysteine in cys-CNF and the formation of its oxidized dimer cystine.

The cysteine-functionalization of CNF was further investigated by elemental analysis, confirming and quantifying the presence of cysteine in the modified CNF material. Elemental analysis indicated that increasing the molar ratio in the reaction did not result in a significant increase in the number of incorporated cysteine (Table 1), obtaining a substitution between 50–60% of the carboxyl groups in the starting material. The cys-CNF material was also analyzed by scanning electron microscopy (SEM) with energy dispersive spectroscopy (EDS), revealing a homogeneous distribution of sulfur on the material surface (Figure S1, Supplementary Materials).

The potential ability of the cys-CNF to modulate the chronic wound environment is expected to depend on the presence of free thiol groups and therefore the cys-CNF was further characterized in terms of free thiol content by the Ellman assay. Results showed that the number of free thiols corresponded to 20–26% of the total sulfur content in cys-CNF (Table 1). The formation of cystine is most probably the reason behind the decrease in thiol groups with respect to the total sulfur content. The presence of cystine in cys-CNF was observed in the NMR spectrum (Figure 2) and the oxidized dimer could have formed during the synthesis step where free cysteines reacted with already immobilized cysteine molecules or during the dialysis steps as a result of the reaction between neighboring immobilized cysteines forming disulfide bonds.

### 3.2. Radical Scavenging Activity

High levels of ROS in chronic wounds have been associated with several adverse effects (e.g., cell damage, inhibition of cell migration, inactivation of metalloproteinase inhibitors, and recruitment of more inflammatory cells into the wound), all contributing to the delayed healing process [30]. Thus, wound dressings with antioxidant properties may modulate the ROS levels in the wound bed and help to reactivate the healing of chronic wounds [31,32].

**Figure 3:**
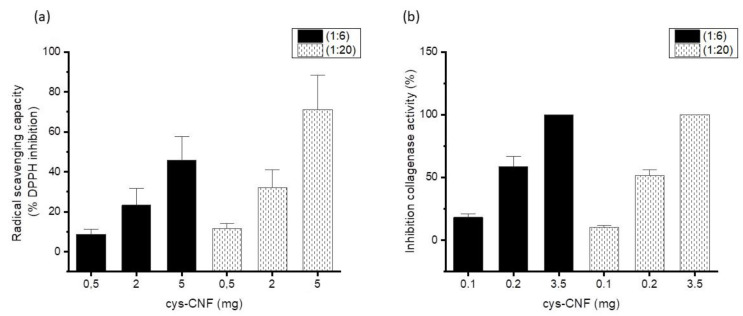
Cys-CNF activity as (**a**) radical scavenging capacity), (**b**) inhibition of collagenase activity. Data are presented as mean ± standard error of the mean of at least three independent experiments. No statistically significant differences were found between the radical scavenging activities of cys-CNF (1:6) and cys-CNF (1:20), nor between the cys-CNF (1:6) and cys-CNF (1:20) collagenase-inhibition capacities.

The antioxidant activity of cysteine is well documented, both in proteins and as free amino acid [25]. Authors have shown that cysteine is one of the most potent antioxidant amino acids, acting as a free radical scavenger and also as a metal chelator [25]. Here, the results indicated that the covalently bound cysteine endowed CNF with radical scavenging activity, with the functionalized CNF material showing a concentration-dependent DPPH inhibition (Figure 3a) while c-CNF did not inhibit DPPH radical activity (data not shown). No statistically significant differences were found between the radical scavenging activities of cys-CNF (1:6) and cys-CNF (1:20).

The antioxidant properties of cellulose-based wound dressings have been previously investigated [33,34,35,36,37]. The ability of the commercially available carboxymethylcellulose-based dressing Aquacel™ to inhibit ROS activity was investigated by Moseley et al., showing that the dressing presented an antioxidant potential most probably based on the restriction of the movement of the free radical (i.e., the ROS molecules can be entrapped into the viscous mesh-network of the dressing) and as a result, they are not able to interact with biological targets [33,34]. Thus, the antioxidant properties of the carboxymethylcellulose-based dressing can be classified as passive, rather than an intrinsic antioxidant capacity of the material. Moreover, when Cullen et al. tested the antioxidant properties of carboxymethylcellulose with the DDPH assay, no activity was found confirming the lack of intrinsic antioxidant activity of the carboxymethylated cellulose [37], a result that is in line with our finding that c-CNF alone lacks free radical scavenging activity. Here, we showed that the covalent functionalization of the cellulose nanofibers with cysteine is a promising approach to obtain a nanocellulose-based dressing capable of directly reacting with free radicals. Another strategy to endow nanocellulose-based dressings with antioxidant properties is to incorporate antioxidant compounds within the fiber matrix, allowing the dressing to act as a drug delivery system [35,36]. This approach presents the challenge of tailoring the drug load and release, while with a cys-CNF based wound dressing, the antioxidant capacity may be easier to control, since the antioxidant properties originate from the dressing matrix itself.

### 3.3. Interactions with the Metalloprotease Collagenase

The elevated activity of metalloproteinases found in chronic wounds causes excessive extra cellular matrix degradation, alteration of cytokine profile, and low levels of growth factors, resulting in the delay or absence of wound closure [22,38]. Several therapies based on the inhibition of metalloproteinase activity have been proposed as promising strategies to treat chronic wounds [22].

We selected collagenase as model metalloproteinase to investigate cys-CNF interactions with this type of proteases. Collagenase incubation with cys-CNF for 24 h resulted in dose-dependent inhibition of the protease activity, reaching total inhibition when the material was assessed at the highest concentration (Figure 3b). To investigate the contribution of protein entrapment within the nanofiber network to the observed decrease in collagenase activity, protein concentration was evaluated after incubating the collagenase with cys-CNF for 24 h. Results showed no protein entrapment with the lowest amount of cys-CNF and only 20–30% of the collagenase was retained when the highest amount of cys-CNF was assessed (Table 2). Thus, the main reason behind the observed decrease in collagenase activity is most probably the protease inactivation rather than just the entrapment of the protein within the fibril network. In this sense, it should also be noted that CNF alone (i.e., c-CNF) did not affect the activity of collagenase (Figure S2, Supplementary Materials).

It is expected that the sequestration of Zn^2+^ ions from the metalloproteinase active site would result in the inhibition of the protease activity [39]. Thus, the Zn^2+^-chelating capacity of cysteine [40] is most probably responsible for the observed decrease in collagenase activity after incubation with cys-CNF. Results of the Zn^2+^ binding assay showed that cys-CNF was able to bind 66 ± 1% of the Zn^2+^ present in a solution incubated with the material in the same conditions as in the collagenase interaction study, while no ion binding was detected with c-CNF. Even though the material interaction with free zinc in solution cannot be directly compared with the access to zinc in a protein’s catalytic center, the above results showed that cysteine did not lose its zinc binding capacity when incorporated into the CNF material.

### 3.4. Material Stability over Time

The instability of thiol-containing compounds toward self-oxidation in the presence of molecular oxygen is well known [41,42] and it could present a challenge for the bioactivity of the cys-CNF material. Even though the complete mechanism is yet to be established, self-oxidation of thiols by molecular oxygen seems to be dependent on pH and on the presence of transition metals that catalyze the reaction (i.e., present due to impurities) [41,42].

**Figure 4:**
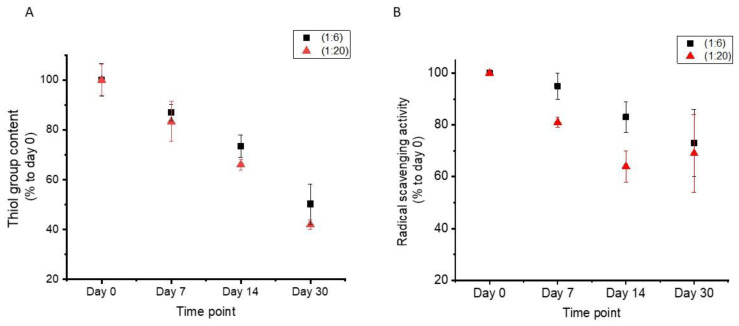
Stability of the thiol group in cys-CNF over time. (**A**) Thiol content measured with the Ellman assay, (**B**) radical scavenging activity assessed with the 1,1-diphenyl-2-picrylhydrazyl (DPPH) assay. Data are presented as mean ± standard error of the mean of at least three independent experiments.

The thiol group content of cys-CNF and its radical scavenging activity were evaluated over a 30 day period. Results showed that when cys-CNF was stored as a gel suspension in close containers at 4 °C, the thiol content decreased over time, showing 40–50% of the initial value 30 days after synthesis (Figure 4A). Even though the capacity of cys-CNF to inhibit the DPPH radical also decreased overtime, the observed loss in radical scavenging activity was less marked than the decrease in thiol content, with the remaining activity after 30 days representing 70% of the initial value (Figure 4B).

Two other storage conditions were evaluated in order to increase the thiol stability over time. To reduce the mobility of the fibers and thus hinder thiol-to-thiol encounters to form disulfide bonds, aerogels were prepared by freeze-drying the cys-CNF suspensions. Additionally, the cys-CNF suspensions were kept under an inert atmosphere to reduce the oxidation rate of the thiol groups. Both cys-CNF aerogels and the cys-CNF in inert atmosphere were more stable than the material stored in suspension (Table 3). Even though the reactivity of the thiol group poses a challenge to the shelf life of cys-CNF, alternative storage conditions such as inert atmosphere or aerogel form are valid approaches to increase the stability of the thiol groups in the cys-CNF material.

### 3.5. Cell Studies

**Figure 5:**
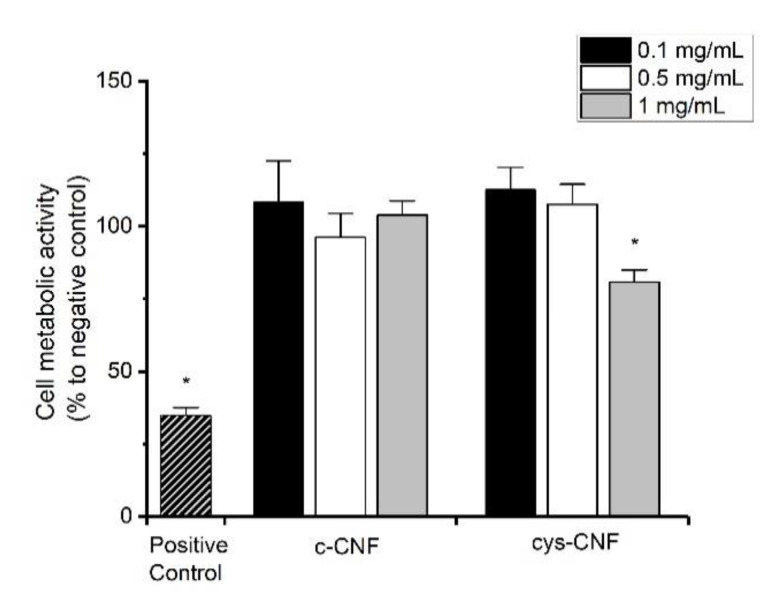
Cell metabolic activity of human dermal fibroblasts after 24 h exposure to cys-CNF and c-CNF suspensions. Data are expressed as percentage of the negative control (non-exposed cells) and presented as mean ± standard error of the mean of at least three independent experiments. The positive control represents cells exposed to 5% DMSO. Significant results compared with the negative control are marked with * (*p* < 0.05).

**Figure 6:**
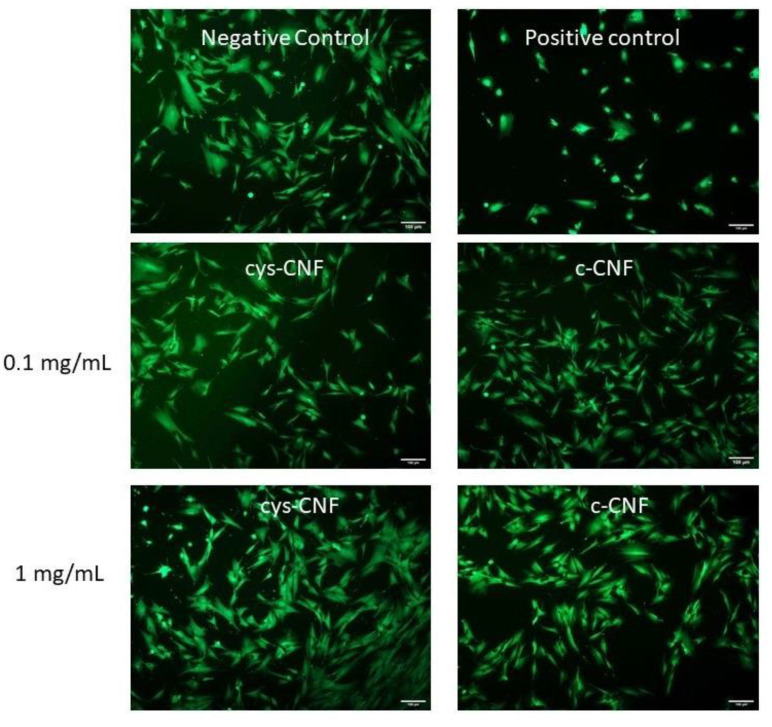
Representative images of calcein-AM stained human dermal fibroblasts after 24 h exposure to cys-CNF and c-CNF suspensions. The negative and positive controls were non-exposed cells and cells exposed to 5% DMSO, respectively. Scale bar represents 100 μm.

If intended to be used as a wound dressing, the safety profile of the thiol-functionalized CNF should be assessed. As a first screening, hDF, a well-established model system to study fibroblast response and wound healing, were selected to evaluate the in vitro toxicity of the cys-CNF material. The cytotoxic effects of cys-CNF (1:20) and c-CNF were evaluated by investigating the effect on cell metabolic activity and by microscopic evaluation of cell viability and morphology after exposure to the materials. Results showed that after 24 h exposure to the CNF materials (concentration range 0.1–1 mg/mL), the cell metabolic activity was above the 70% cytotoxicity limit described by the ISO standard 10993-5 [43] (Figure 5). However, the highest concentration of cys-CNF induced a statistically significant decrease in cell metabolic activity compared with the negative control. Microscopy observations showed that the number of viable cells after exposure to the materials was comparable to the number of cells present in the negative control (non-exposed cells). Furthermore, the cell morphology was not affected by the exposure to increasing concentrations of cys-CNF or c-CNF, with cells maintaining the characteristic elongated shape of fibroblast cells in contrast with the round-shaped cells found in the positive control (Figure 6).

Overall, cys-CNF did not induce toxic effects on hDF when tested at a concentration up to 0.5 mg/mL, nor did the starting material c-CNF. These results confirm the safety profile of nanocellulose fibers when in contact with biological systems, as previously investigated with different functionalized CNF materials and diverse cell models [44,45,46,47]. Future studies should investigate the in vitro wound healing capacity of cys-CNF in terms of dermal and epidermal cell response (proliferation, migration, and differentiation).

## 4. Conclusions

In the present study, we showed that, by a well-known chemical approach, the amino acid cysteine was covalently incorporated into CNF endowing CNF with bioactivity toward the modulation of the chronic wound environment. The cys-CNF material presented a dual action in vitro: inhibition of metalloproteinase and radical scavenging activity, which are expected to contribute to the restitution of physiological conditions in the wound bed and promote the resolution of hard-to-heal wounds.

## Figures and Tables

**Table 1:** Cysteine content, degree of substitution, and free-thiol content of the cys-CNF materials obtained using two different molar reaction ratios.

Molar Reaction Ratio ^1^	Cysteine Content (mmol/g CNF) ^2^	Degree of Substitution ^3^	Thiol Content (mmol/g CNF) ^4^	Thiol/Sulfur (%)
1:6	0.71 ± 0.07	0.54 ± 0.05	0.14 ± 0.02	20
1:20	0.78 ± 0.04	0.60 ± 0.05	0.20 ± 0.03	26

^1^ COOH:cysteine. ^2^ Determined by elemental analysis of sulfur content. ^3^ Number of cysteine (mmol/g CNF) per number of carboxyl groups in the starting material c-CNF (1.3 mmol/g CNF). ^4^ Determined by the Ellman assay.

**Table 2:** Entrapment of collagenase after incubation with the cys-CNF suspension (3.5 mg dry content).

Cys-CNF Sample	Collagenase Entrapment (% of Control)
(1:6)	20 ± 5
(1:20)	30 ± 5

**Table 3:** Remaining thiol content (%) in cys-CNF after 30 days of storage at different conditions.

Cys-CNF Sample	Suspension (Air)	Aerogel	Inert Atmosphere (N_2_)
(1:6)	49 ± 13	67 ± 19	105 ± 4
(1:20)	43 ± 8	89 ± 11	76 ± 4

## Data Availability

The data presented in this study are available on request from the corresponding author.

## Supplementary Materials

The following are available online at https://www.mdpi.com/2073-4360/13/2/249/s1, Figure S1. (A) Representative SEM image of cys-CNF film with (B) its corresponding scanning of sulfur by SEM-EDS. Scale bar 1 μm; Figure S2. Collagenase assay kinetics. Representative data of the collagenase activity after incubation with cys-CNF and c-CNF. Control refers to collagenase incubated without CNF materials.
