# Supplementary material for: In Vitro Investigation of Thiol-Functionalized Cellulose Nanofibrils as a Chronic Wound Environment Modulator
Source: Polymers (Basel). 2021 Jan 13;13(2):249. doi: 10.3390/polym13020249 (PMC7828681; doi:10.3390/polym13020249)
Supplement: Supplementary file 1 [file polymers-13-00249-s001.pdf]

Supplementary Materials

In Vitro Investigation of Thiol-Functionalized Cellulose Nanofibrils as a Chronic Wound Environment Modulator

Anna Blasi-Romero [1], Carlos Palo-Nieto [1], Corine Sandström [2], Jonas Lindh [1], Maria Strømme [1] and Natalia Ferraz [1,*]

[1] Nanotechnology and Functional Materials, Department of Material Science and Engineering, Uppsala University Box 35, 75103, Uppsala, Sweden; anna.blasi@angstrom.uu.se A.B.R.), carlos.nieto@angstrom.uu.se (C.P.N.), jonas.lindh@angstrom.uu.se (J.L.), maria.stromme@angstrom.uu.se (M.S.); natalia.ferraz@angstrom.uu.se (N.F.)

[2] Department of Molecular Sciences, Swedish University of Agricultural Sciences, Box 7015, 75007, Uppsala, Sweden; corine.sandstrom@slu.se (C.S.)

* Correspondence: natalia.ferraz@angstrom.uu.se (N.F.)

1. Methods

1.1. Scanning Electron Microscopy/Energy Dispersive Spectroscopy (SEM/EDS)

The distribution of cysteine on the nanofibers was investigated using scanning electron microscopy (SEM) imaging together with energy dispersive spectroscopy to detect sulfur. Cys-CNF suspensions were air-dried, placed on a carbon stub, and coated with a thin layer of gold/palladium with a sputter coater Polaron SC7640 sputter coater (Thermo VG Scientific) to be imaged using a Zeiss LEO 1550 SEM with SE2 detector and an energy dispersive detector EDS (Carl Zeiss Microscopy, Oberkochen Germany).

2. Results

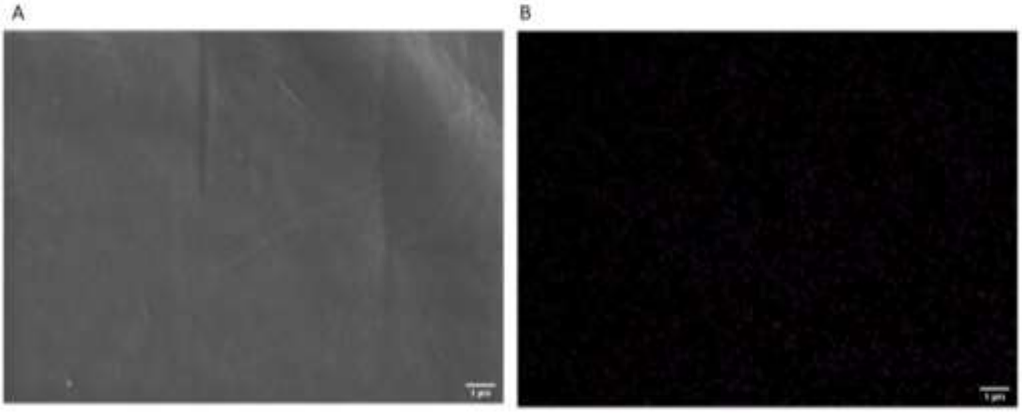

Figure S1. (**A**) Representative scanning electron microscopy (SEM) image of cys-CNF film with (**B**) its corresponding scanning of sulfur by SEM-energy dispersive spectroscopy (EDS). Scale bar 1 μm.

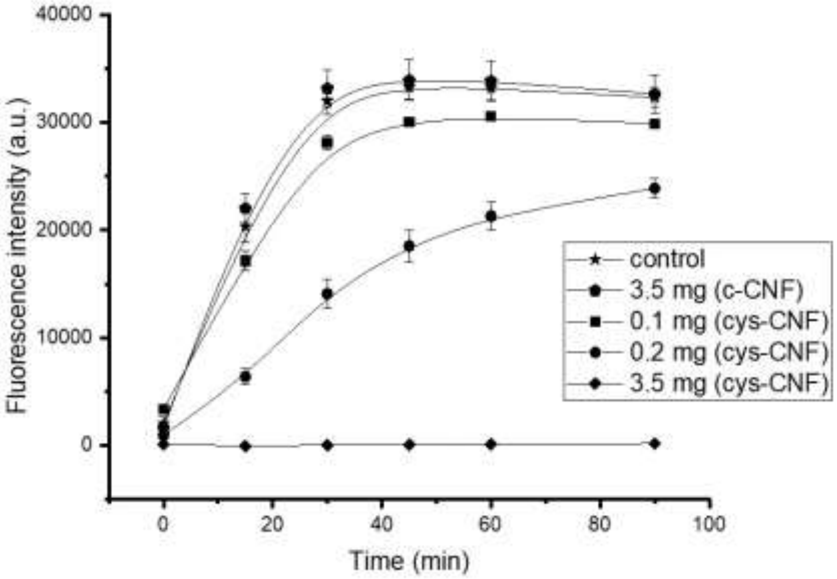

Figure S2. Collagenase assay kinetics. Representative data of the collagenase activity after incubation with cys-CNF and c-CNF. Control refers to collagenase incubated without CNF materials.
